# Utility-value change and the role of emotional cost in video-based learning: a matter of student teachers’ interpretation of experience

**DOI:** 10.3389/fpsyg.2023.1166921

**Published:** 2023-07-26

**Authors:** Mathias Dehne, Alexander Gröschner

**Affiliations:** Faculty of Social and Behavioural Sciences, Friedrich Schiller University Jena, Jena, Germany

**Keywords:** utility-value intervention, emotional cost, video-based learning, latent change analysis, teaching practicum

## Abstract

Motivational and emotional characteristics influence teachers’ reflections on video clips from their own teaching. However, utility values and the role of emotional cost have not been considered yet in video-based research in teacher education. In the present study, 102 student teachers were randomly assigned to an intervention group (IG) with video-based documentation of a lesson and systematic writing assignment or to a control group (CG) with protocol-based documentation of a lesson and writing assignment. Multigroup latent change score analysis indicated that IG participants, on average, showed a 0.52 *SD*s higher increase in utility values than the CG three months after the teaching practicum. Emotional cost was negatively related to baseline utility-value scores but predicted latent change scores positively after the writing assignment. The study adds to the current repertoire of video-based learning opportunities by providing a systematic writing assignment targeting student teachers’ interpretation of experiences to leverage motivation.

## 1. Introduction

Utility values are typically addressed by a scaled-up wise intervention approach known as utility-value interventions (Gaspard et al., 2021). When students struggle in courses, psychological processes might underlie that “interfere with optimal academic functioning” (Harackiewicz and Priniski, 2018, p. 410) and “often act as key levers … that give rise to social problems” (Walton, 2014, p. 80). Drawing on situated expectancy-value theory (SEVT; Eccles and Wigfield, 2020), this study explored the utility-value change of student teachers reflecting by video vs. student teachers reflecting by a protocol on a lesson taught during their teaching practicum. Ample research has demonstrated the effectiveness of video-based interventions in terms of enhancing student teachers’ classroom-related outcomes, such as “noticing” or “reflecting” on teaching and learning (Korthagen, 2010; Gaudin and Chaliès, 2015; Kleinknecht and Gröschner, 2016; König et al., 2022). Although previous research has stressed that higher utility values can be expected for video-based compared to text-based reflections (*cf.* Brouwer et al., 2017), studies concluded that video does not automatically activate emotional and motivational processes (Kleinknecht and Schneider, 2013). Neither does simply viewing video guarantee teacher learning (Gaudin and Chaliès, 2015). In contrast, research has not yet implemented interventions targeting student teachers’ motivation in complex video-based learning environments with a higher cognitive load than protocol-based methods induce (Weber et al., 2023). Consequently, the role of utility values remains unexplored (Dehne et al., 2018; Nickl et al., 2023). Drawing on this body of research, the design of augmented learning environments regarding student teachers’ motivational and emotional characteristics is an important prerequisite to enhancing teacher learning with video.

In this study, we tested a new intervention approach in line with SEVT and previous utility-value-like interventions in order to promote student teachers’ utility values for reflecting on teaching and learning (*cf.* Harackiewicz et al., 2012). Student teachers had to relate their video-based or text-based reflection to their achievement-related experiences and interpretations thereof (Brouwer et al., 2017). Additionally, we investigated the influence of emotional cost on changes in student teachers’ utility values in video-based learning. Control-value theory, as a well-known framework on achievement emotions, has pointed out that negative outcome emotions such as anxiety and hopelessness are aroused by a lack of control and doubts about the certainty of success and negatively relate to achievement (Pekrun, 2006). Regarding video-based interventions, research has referred to the negative emotional arousal for video-based interventions when they make use of participants’ video clips of their teaching (e.g., Kleinknecht and Schneider, 2013; Chan et al., 2018), whereas the learning outcome will be influenced negatively (Charalambous et al., 2022). By investigating motivational and emotional characteristics with the conceptual lens of SEVT, our study will provide new knowledge to promote student teachers’ video-based learning.

### 1.1. Situated expectancy-value theory

**Figure 1:**
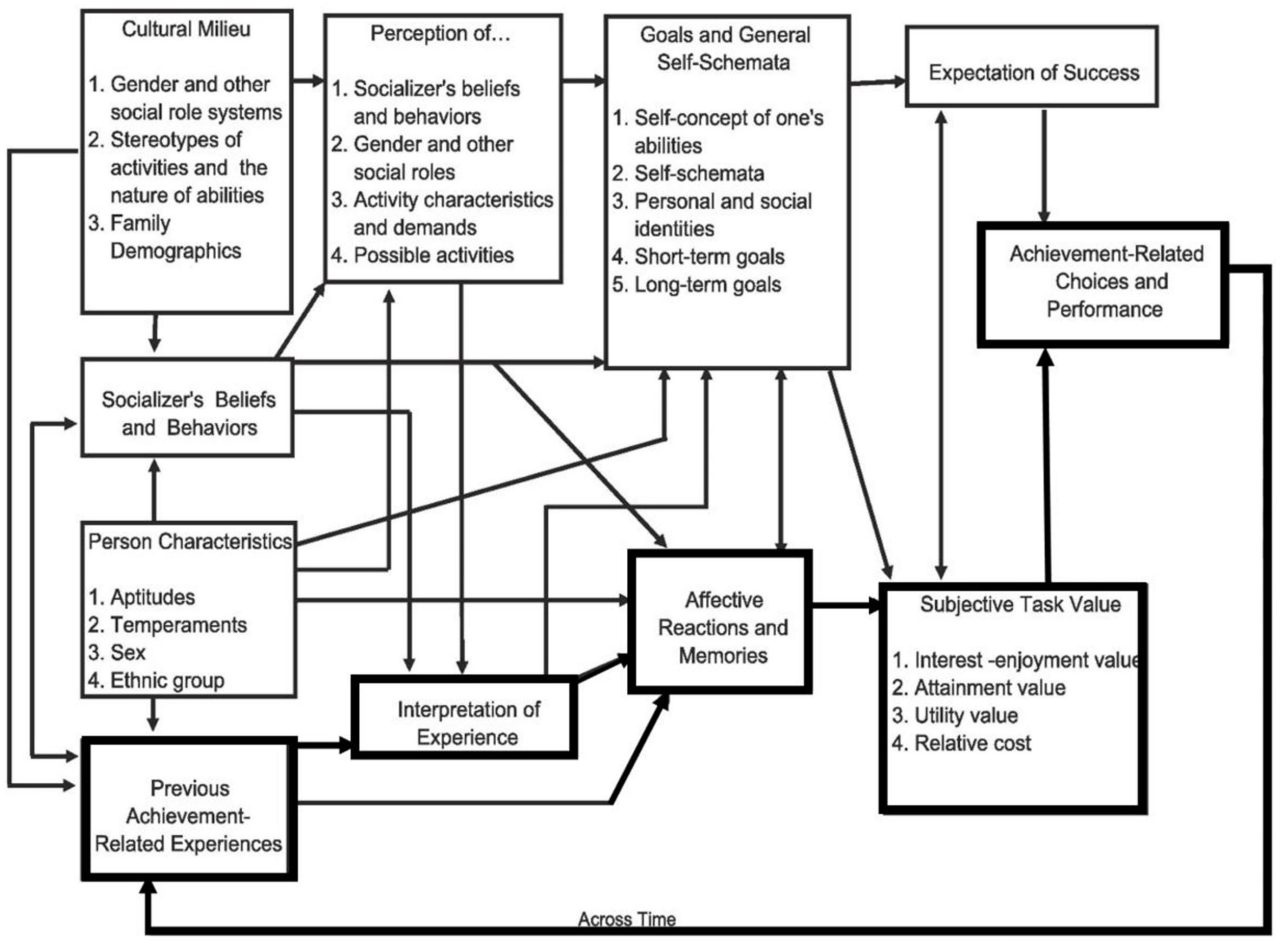
Situated expectancy-value model of achievement-related choices and performance. Bold = Paths underlying the intervention. Reprinted from Contemporary Educational Psychology, Vol 61, Jacquelynne S. Eccles & Allan Wigfield, From expectancy-value theory to situated expectancy-value theory: A developmental, social cognitive, and sociocultural perspective on motivation, Page 2, Copyright (2020), with permission from Elsevier.

SEVT constitutes a comprehensive lens for investigating motivation and its relationship to various academic outcomes, such as effort and course grades, in various academic settings. In being ample regarding the background characteristics and the ontogeny of achievement motivation, the model provides a broad range of paths to be harnessed in interventions (see Figure 1). However, when the model is traditionally considered in psychological research, expectancy beliefs (“Can I do this task?” with its self-efficacy-like implications) and values (“Why should I do this?” with personality, intrinsic, extrinsic, and cost implications) are differentiated as predictors of achievement-related choices among students (Eccles and Wigfield, 2002, 2020; Eccles, 2005). In this regard, utility value reflects the relevance to future goals (Eccles, 2005). Cost marks the fear of failure or perceived psychological threat associated with a task (Song et al., in press). When students value a task and believe they can succeed, they are more likely to take on a challenging task, as negative emotions will be lowered (Pekrun, 2006; Harackiewicz et al., 2012). In this regard, the critical role of cost perceptions as part of SEVT has been widely remarked upon, although it has received relatively little research attention (Rosenzweig et al., 2020). Cost perceptions negatively affect students’ learning outcomes. For instance, university students with higher perceptions of cost show stronger intentions to leave their STEM major (Perez et al., 2014). Flake et al. (2015) focused on a more sophisticated differentiation of the cost component in task effort, outside effort, loss of valued alternatives, and emotional cost. Recently, Song et al. (in press) have critically investigated emotional cost and anxiety regarding their mutual relevance in students’ experiences. Both constructs provided insufficient evidence for structural and discriminant validity (i.e., high cross-loadings and high factor correlations). Furthermore, both constructs showed an overlap when predicting final exam scores in an introductory biology course at the university with almost the same magnitude. These findings support our assumption that emotional cost could be related to video-based learning during a teaching practicum, as previous research has pointed out anxiety as one of the most prevalent achievement emotions when using videos of one’s teaching (e.g., Chan et al., 2018).

The exclusive focus on expectancies and values as one part of the model in the past decades has prompted Eccles and Wigfield (2020) to refine the model and refer more specifically to its situated components. Consequently, they referred to facets included in the social and experiential background as well as surrounding interpretations of experiences as the “often-neglected” left and middle parts. Although the components have not been the focus, the experiential background and achievement-related interpretations in the SEVT model frame personal characteristics, the enculturation, and socialization of a person’s motivational beliefs and illustrate how cognitive processes mediate the effects of social and experiential background with regard to expectancies, values, and the self-concept of ability (Eccles and Wigfield, 2020; *cf.* Tschannen-Moran et al., 1998).

### 1.2. The psychology of utility-value interventions

Reasons for learning more abstract contents and higher-order processes are typically not obvious (Wentzel and Brophy, 2014). Likewise, this circumstance applies to more immersive video-based reflections in a teaching practicum (*cf.* Beck and Kosnik, 2002). Inter-individual variability regarding the learning outcomes and difficulties student teachers experience will result from video-based learning activities, as a study on ambitious teaching pointed out (Charalambous et al., 2022). Consequently, teacher educators are requested to design learning environments thoroughly and consider student teachers’ heterogeneous backgrounds regarding their emotions and motivation as they matter for their learning outcomes and professional competence (Vermunt and Endedijk, 2011; Kunter, 2013; Klassen et al., 2020). Kleinknecht and Schneider’s (2013) emphasis that video-based reflections do not automatically motivate (student) teachers and will thus not automatically lead to learning gains without regarding their learner characteristics captures a major requirement for teacher learning in any learning environment (e.g., Vandewaetere et al., 2011; Bardach et al., 2022). To date, video-based research lacks interventions on student teachers’ psychological characteristics (e.g., motivation or personality) that would contribute knowledge about ways to scaffold them in video-based tasks.

During the last decade, research employing utility-value interventions provided substantial evidence for their effectiveness in fostering student outcomes, such as interest, utility values, retention, course grades, or continuation to the next course, mainly in STEM fields (Hulleman and Harackiewicz, 2009; Lazowski and Hulleman, 2016; Harackiewicz and Priniski, 2018; Hulleman and Harackiewicz, 2021). They have been applied in such diverse contexts like mathematics classrooms in school (Gaspard et al., 2015a), introductory biology courses in universities (Canning et al., 2018), and for parents to foster adolescents’ career orientation, career support, and the importance of career support (Piesch et al., 2019). Typically, social-psychological utility-value interventions use essay-based approaches with only little effort to implement. Here, students are asked to relate course content in a personal (e.g., by pronoun use), specific (reg. Course content and its relevance for an activity, hobby, etc.), and context-relevant manner (reg. Course content and everyday life or future career goals). According to Hulleman and Harackiewicz’ (2021) logic model, these aspects add up to an individual’s intervention fidelity; they are part of a process of raising students’ perceived utility and success expectations and lowering their perceived cost. In turn, this process contributes to co-occurring psychological (e.g., developing an enduring interest or becoming more actively involved in a task) and behavioral (e.g., engagement or performance) mechanisms. Finally, outcomes such as grades, persistence, or career intentions are fostered.

By harnessing utility values in this set of interventions, STEM students in an introductory undergraduate biology course were more likely to enroll in the second course (Canning et al., 2018). Concerning the timing, students with a history of poor achievement benefitted most from a utility-value intervention at the beginning of the semester. In contrast, higher-performing students benefitted most by completing a utility-value task at the end of the semester to enroll in the second course. However, Canning et al. (2019) found some unintended consequences: struggling university students lost interest and perceived less utility. In a similar vein, the essay’s length and quality (i.e., using simpler words) decreased for struggling students in one intervention assignment. Another strand of research has focused on whether an essay-based intervention design works best, showing equal or more favorable results for quotation-based approaches where students are asked to evaluate quotations from former students regarding their personal relevance (Gaspard et al., 2015a, 2021). Although the results are promising in terms of transferring experiences into students’ mindsets (*cf.* Eccles and Wigfield, 2020), these findings are limited to high school students.

In an intervention approach to career orientation with parents, utility-value quotes from former students and parents were presented on a website. It was intended to address students’ interpretations of experiences more directly by socializers’ beliefs (Piesch et al., 2019). However, the study by Piesch et al. found no positive intervention effects on either students’ or parents’ utility values or career support and orientation. Thus, the promising results from Harackiewicz et al. (2012) harnessing parental behaviors (and thus also adolescents’ perceptions of their behaviors) for students’ interpretations of experiences were not replicated. Harackiewicz’s team mailed brochures to parents and used a website over a 15-month period that led students to take significantly more mathematics and science classes during their last two years of high school compared to a control group. Eccles and Wigfield (2020) acknowledged this as a step in the right direction (i.e., focusing on the “often-neglected middle part” of the SEVT model) “by introducing new experiences into students’ school and family settings, thus acknowledging the role of experience” (p. 8). However, most utility-value interventions do not fully cover experiential backgrounds. Especially for the teaching practicum, it is argued that student teachers value it most when experiences are being analyzed, for instance, as part of the course (Kleinknecht and Gröschner, 2016). This raises the question: What happens if student teachers’ interpretations are addressed directly in interventions (linked to affective memories and indirectly to utility values) after an achievement-related experience?

Similar to utility-value interventions, it could be assumed that a cost-effective essay-based approach implemented during the semester might be utilized to address student teachers’ interpretations of experiences more specifically. In this sense, the interpretation-of-experience box (Figure 1, in bold) could be understood with Tschannen-Moran et al.’s (1998) cyclical model of teacher efficacy. Cognitive (interpretative) processes mediate the influence of sources of teacher self-efficacy (e.g., mastery experience or verbal persuasion) on the task and ability analysis and, as a result, indirectly shape teachers’ sense of efficacy. Thus, simply introducing the relationship of a certain topic to one’s daily life or career goals is not to be seen as promising in enhancing student teachers’ motivation. Rather, how they react and attempt to overcome obstacles in the future and whether they are capable of doing so is the key to maintaining motivation for certain topics (*cf.* Kunter, 2013). In the following section, we will liken common motivational and emotional conceptualizations for student teachers’ video-based learning with utility values and emotional cost.

### 1.3. Video-based learning and emotional-motivational processes

Ample research has focused on the motivational and emotional facets of (student) teachers’ video-based learning (Goldman, 2007; Seidel et al., 2011; Kleinknecht and Schneider, 2013; Gaudin and Chaliès, 2015; Kleinknecht and Gröschner, 2016). However, the conceptualizations of different facets remain heterogeneous (Dehne et al., 2018). Video, in this context, is regarded as a powerful tool as it provides performance in action and can be repeatedly viewed and analyzed from different perspectives (Brouwer et al., 2017). During a teaching practicum, video-based tools act as a “third space” where practitioner and academic knowledge are brought together in new hybrid ways to enhance student teachers’ learning (Zeichner, 2010). Research on teachers’ video-based learning (e.g., Seidel et al., 2011) often characterizes emotional and motivational processes by the terms “resonance” and “immersion.”

#### 1.3.1. The concepts of resonance and immersion

*Resonance* shows a direct relation to the concept of utility value. It captures how a video-based learning tool is connected to the viewer’s situation (Goldman, 2007). More specifically, resonance can be conceptualized in terms of the relevance for student teachers’ future work in the teaching profession (Dehne et al., 2018). Hence, the resonance one perceives when watching a video points to the facet utility for job (*cf.* Gaspard et al., 2015b), if the video is guiding one’s focus on his/her instructional practices (Seidel et al., 2011). Consequently, research has stressed the importance of utility values for (student) teachers’ intention to use technology in the classroom or their frequency of technology integration (Teo, 2009; Backfisch et al., 2021). However, research has yet largely missed introducing the concept of utility value to student teachers’ video-based learning.

In contrast, Goldman (2007) refers to *immersion* as a form of deep-level engagement or emotional involvement in the topic. While it contains positive facets such as excitement (Seidel et al., 2011), the other side of the coin might be a feeling of shame as a direct result of videotaping, having to select, and later watching own—potentially unpleasant—classroom sequences (Chan et al., 2018). Richards et al. (2021) have pointed to this complex interplay when teachers generate video clips for teacher professional development programs. While generating videos already guides teachers’ focus to student thinking, the complexity of the whole process makes less favorable outcomes possible, especially in diverse settings. Dehne et al. (2018) have shown that sustaining these efforts requires increased support in teacher education. Emotional cost is a task value facet described by SEVT (*cf.* Gaspard et al., 2015b; Part et al., 2020) and is directly related to immersion. The concept is helpful in refining the understanding of the process of immersion, which has been regarded positively as a motivational reference for teachers’ involvement in the video-based task (Seidel et al., 2011). As emotional cost refers to the broader motivational value of a task (*cf.* Part et al., 2020), it might enrich conclusions for value-based interventions in heterogeneous learning communities.

Reviews on (student) teachers’ video-based learning (Gaudin and Chaliès, 2015; Santagata et al., 2021) referred to studies where teachers watched videos of their own teaching versus videos of peers or unknown teachers (Seidel et al., 2011; Kleinknecht and Schneider, 2013; Chan et al., 2018) and where video-based conditions were compared to protocol-based conditions (Moreno and Valdez, 2007; Kleinknecht and Gröschner, 2016; Prilop et al., 2019) as significant research strands. A large body of research has sought interrelations between the degree of emotional-motivational activation when (student) teachers watch videos of their own versus videos of others teaching. Following, we refer to exemplary findings that provide insight into individual learning with video and emotional-motivational processes.

#### 1.3.2. Literature review

Seidel et al. (2011) found that teachers who watched videos of their own teaching showed higher levels of immersion and resonance compared to teachers watching videos of others’ teaching. In addition, when teachers were asked whether they found using video motivating, those using their own videos again showed higher scores. Against this backdrop, Kleinknecht and Schneider’s (2013) study investigated the activation of emotional-motivational processes for teachers reflecting on videos of their own compared to others’ teaching. The study showed no significant differences in immersion or resonance in teachers’ comments on the respective videos. However, a tendency was found that teachers in the “own video group” showed more resonance with their practice. Counterintuitively, they found that participants in the “other video group” showed significantly more emotional expressions and a tendency toward more negative emotions. This finding is in stark contrast with Chan et al. (2018). They found that student teachers experienced more frequent negative emotions such as anxiety or shame when watching videos of their own teaching compared to fewer negative and more positive emotions when watching videos of their peers. The authors concluded that although reflecting own practices retrospectively and apart from cognitive and emotional involvement in the teaching situations in which they occur, a variety of negative emotions is elicited. Whether the difference compared to Kleinknecht and Schneider’s (2013) study dates back to different stages of experience (in- vs. pre-service) has not been reflected. One assumption was that certain emotions when watching own teaching for the first time are more likely or that these emotions result from unrealistic and unmet expectations.

Regarding research that compared video-based with protocol-based reflections, Prilop et al. (2019) used the setting of a teaching practicum in teacher education to investigate changes in teacher self-efficacy and constructivist beliefs. However, self-efficacy was fostered in either group, and constructivist belief did not decrease for the video-based reflections but for protocol-based conditions. Dehne et al. (2018) showed a similar finding regarding student teachers’ expectancy for success and related it to overall mastery experiences in the teaching practicum. Additionally, the study by Prilop et al. (2019) showed that protocol-based reflections led to increases in more traditional teaching beliefs. In another study, Koehler et al. (2005) found some beneficial effects of video on college students’ emotional engagement, positive affect, or interest engagement compared to text-based approaches. The authors linked this finding to information typically unavailable in texts (e.g., information about mood or tone). However, they noted that the effects of videos are not ubiquitous and largely depend on the context for which researchers have developed structured viewing guides in the field of teacher education (Brouwer et al., 2017). In essence, protocol-based approaches offer better possibilities for comparing the merits of video-based tools since video as a medium shows a stark contrast with additional sources of information (concrete contexts, audio, etc.) not available in a protocol format (*cf.* Prilop et al., 2019).

A few limitations arise from the reviewed literature. First, more robust analyses and larger samples must be considered in educational research to verify findings on (student) teachers’ motivation and emotions, as case study designs were oftentimes adopted. Second, the context of the studies must be considered. All studies attempted to harness teachers’ learning (e.g., noticing or reflecting) with videos. However, virtually none of them directly addressed what Kleinknecht and Schneider (2013) called for—to make use of more prearrangements or scaffolding, which is especially necessary when reflecting on their own videos (see also Brouwer et al., 2017). As resonance has been described as the extent to which teachers are able to relate a video to their own experience (Goldman, 2007) and has yielded mixed results with a tendency to be more activated when reflecting on their own videos (e.g., Seidel et al., 2011), our intervention targeting student teachers’ interpretations of experiences shows a promising and easy-to-implement approach to foster student teachers’ utility beliefs in the context of video-based studies.

### 1.4. The present study

In the present intervention study, we focused on the extent to which a video-based learning tool in combination with a structured essay-based writing assignment leads to changes in student teachers’ utility values (*cf.* Kleinknecht and Schneider, 2013; Brouwer et al., 2017). Additionally, we focused on student teachers’ emotional cost as a predictor of utility-value change to capture difficulties that student teachers perceive when using videos of their own teaching (*cf.* Chan et al., 2018) with quite plenty of simultaneous tasks to execute when videos are generated (Richards et al., 2021). The combination of video-based learning in teaching practicums has been described as a promising conducive context as student teachers have contact with real students in authentic classroom situations, whereas the video setting enables them to collaboratively, supportively, and with discipline reflect on examples of their own teaching (Charalambous et al., 2018, 2022). Finally, by contrasting the effects of this intervention with a group using a protocol-based approach, we referred to findings emphasizing benefits concerning affective processes and transfer of learning outcomes (Koehler et al., 2005; Moreno and Valdez, 2007; Kleinknecht and Gröschner, 2016; Prilop et al., 2019; Weber et al., 2023). This study will contribute new evidence to the role of utility values and emotional cost for, but not exclusively, video-based learning in teacher education.

### 1.5. Research questions and hypotheses

The following research questions were investigated:

(*RQ 1*) How do utility values change in a group with video-based documentation of a lesson and systematic writing assignment (IG) compared to a group with protocol-based documentation of a lesson and unsystematic writing assignment?

*We expect that harnessing student teachers’ interpretation of experiences in the IG will increase latent difference scores compared to CG.* (*Hypothesis 1.1*)

*Furthermore, we expect significant interindividual variability in IG participants’ latent difference scores and negative bivariate associations between their initial utility values and their latent difference scores since the writing assignment targets student teachers perceiving video-based learning as challenging.* (*Hypothesis 1.2*)

(*RQ 2*) What effects arise concerning the influence of emotional cost on changes in utility values?

*We hypothesize that latent difference scores in the IG are positively affected by emotional cost. More relevant, we assume, in contrast, negative associations regarding their initial utility values. This hypothesis was formulated with respect to the specifics arising if experiences are addressed systematically so that struggling student teachers can benefit.* (*Hypothesis 2*)

## 2. Materials and methods

### 2.1. Sample

A full cohort of 102 student teachers (*M*_age_ = 22.73, *SD* = 3.23; 63.2% female) was enrolled in the study and gave their informed consent to participate. The Ministry of Education, Youth, and Sport granted ethical approval for the study. In their third academic year, student teachers attended a five-month teaching practicum in a German teacher education program for secondary education in all subjects. To answer our research questions, we implemented a pre-test-post-test control group design. Participants were randomly assigned to an intervention group receiving a systematic SEVT-based writing assignment and video-based documentation intervention (*n_IG_* = 51) or an unsystematic writing assignment and protocol-based documentation (*n_CG_* = 51). Student teachers spent four days a week in different placement schools to which *n* = 2–3 preservice teachers were randomly assigned and one day on campus during the practicum.

### 2.2. Intervention

#### 2.2.1. Lesson documentation and writing assignments

**Figure 2:**
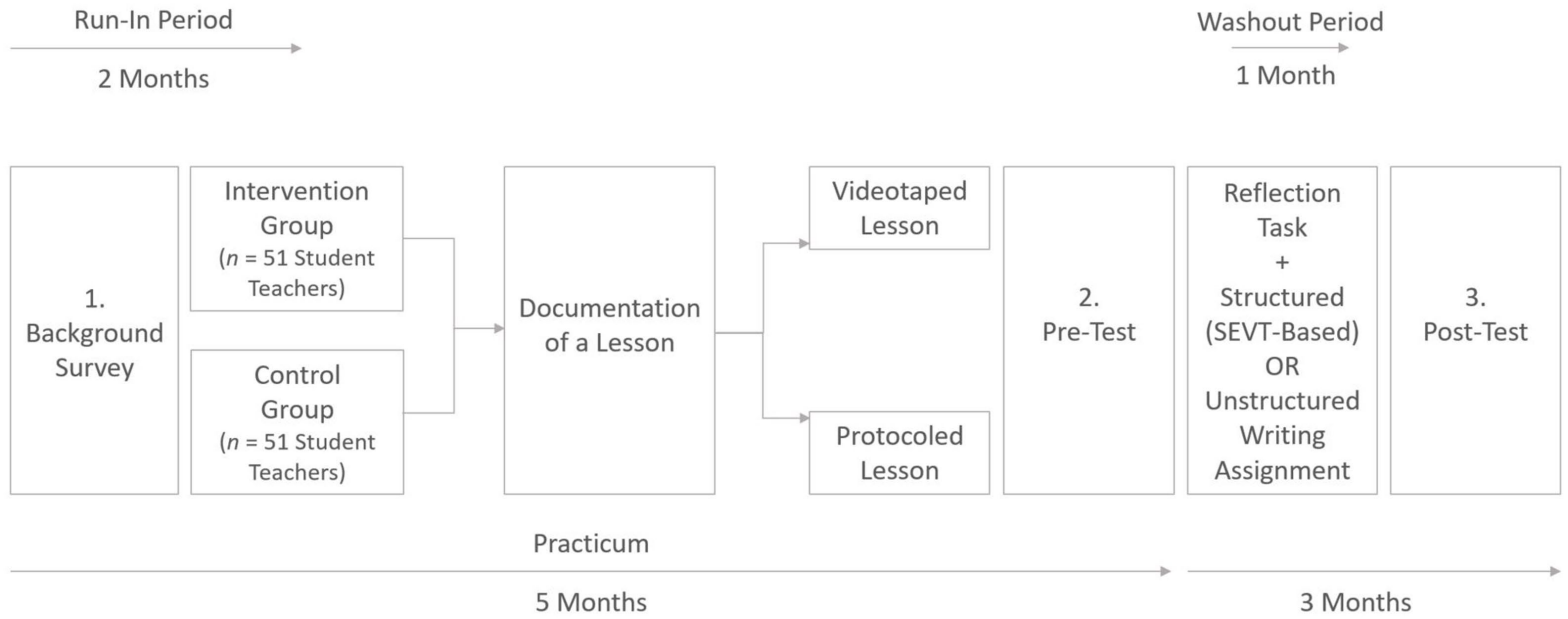
Study design.

A schematic representation of the study is depicted in Figure 2. Student teachers had a passive run-in period after the first course in which they filled in a background survey. During this period, student teachers observe teaching and make their first experiences under mentorial observation. As part of the accompanying courses in educational sciences that started two months later, student teachers in the IG and CG were advised to plan a lesson with the scope of academically productive talk (see O’Connor et al., 2017). Student teachers uploaded a lesson plan that was further revised based on coursework. In the mid of the practicum, student teachers in both groups held a lesson based on their lesson plan in the placement school. IG participants videotaped the lesson and were provided technical support by a fellow student at the same placement school. In turn, CG participants’ lesson was protocoled in written form by a fellow student who sat in. Finally, student teachers were instructed in the last course session (end of the teaching practicum) to reflect on the lesson as the major objective for their final papers that were credited with 10 ECTS. For this purpose, they chose a research question and consequently had to base their reflections on the lesson on a standardized approach comprising a three-step analysis of description, explanation, and suggesting alternatives (Seidel and Stürmer, 2014; Kleinknecht and Gröschner, 2016).

IG participants had to provide an additional ½ of a page in which they reflected on their achievement-related experiences (Eccles and Wigfield, 2020). Student teachers in both groups were requested to reflect on a self-chosen topic covered in the courses using course materials and notes. In line with Hulleman and Harackiewicz (2021), the essays of IG participants had to comprise

1. Personalized narratives on strengths, challenges, and how they overcame obstacles;
2. Strongly contextualized information on findings in the video analysis and theoretical claims made in their papers;
3. Implications for their future professional practice with information on what they would like to improve based on the video excerpts

The intervention approach, therefore, highlights aspects of Rosenzweig et al.’s (2020) cost-reduction intervention that aimed students by quotations of former physics students to reflect challenges positively to make them seem less costly. The control group, in contrast, was requested to provide ½ of a page with unstructured information regarding a topic covered in the accompanying courses. Information should be structured in a meaningful way by using own words without further requirements. The essay-based writing assignments were to be fulfilled within one month (washout period) after the pre-test.

#### 2.2.2. Randomization test and manipulation check

Participants in the two groups did not significantly differ in gender (*χ*^2^[1] = 3.50, *p* = 0.061), age (*t*[81] = 0.32, *p* = 0.747), lessons taught in the placement school (*t*[41.85] = 1.14, *p* = 0.261) or the time (in minutes) per week that mentor teachers supported student teachers (*t*[75] = −1.26, *p* = 0.212). We conducted a manipulation check when the teacher educators graded the final papers. Therefore, teacher educators checked whether the essay comprised the three above-mentioned aspects (see Chapter 2.2.1). The quality of the essays was not coded. All participants in both groups complied with the lesson documentation and submitted their final papers. Only one participant in the IG did not provide the additional writing assignment.

### 2.3. Data collection and operationalization

Utility values were assessed with a questionnaire at the end of the teaching practicum in the closing session of the accompanying course (pre-test) and three months later (post-test). Emotional cost was assessed only at the pre-test. The pre-test, hence, assessed student teachers’ emotional-motivational beliefs in terms of the video-based vs. protocol-based reflection, whereas the post-test highlights how utility values changed after the writing assignments. In addition, a background survey captured student teachers’ characteristics before the teaching practicum while also comprising the utility-value scale. Utility values, reflected by the facet utility for job, and emotional cost (see Appendix A Table A1 for all questionnaire items), were assessed on an instrument developed by Gaspard et al. (2015b). This instrument aimed for a finer-grained utility value and cost differentiation and has been applied in other interventions (e.g., Rosenzweig et al., 2020). We slightly modified the items in a content-specific way (see Dehne et al., 2018, for the validation of the instrument) and evaluated the scale reliability ρ for utility values and emotional cost (see Table 1).

### 2.4. Data analysis

**Figure 3:**
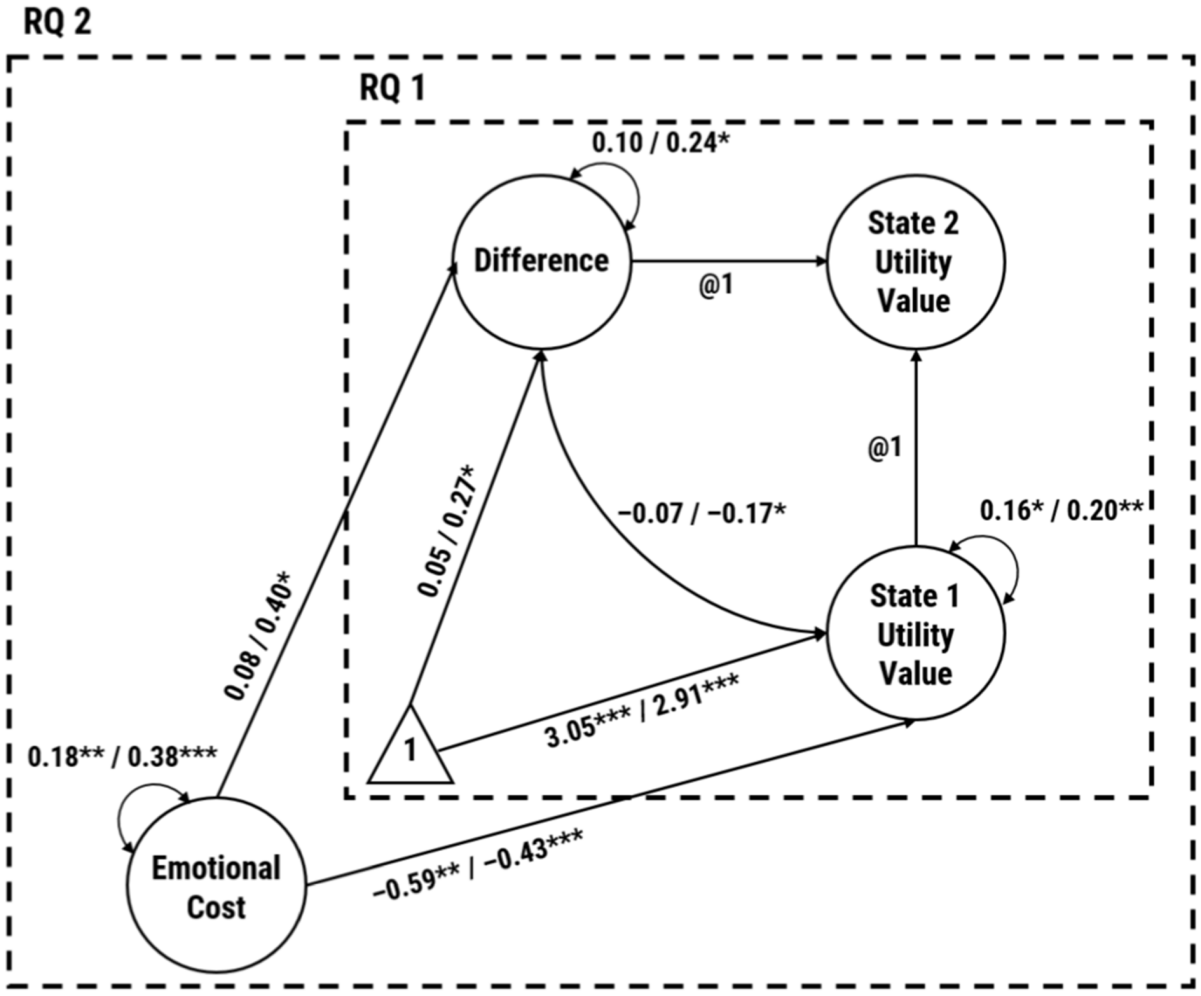
Multigroup latent change score model for testing unconditional and conditional utility-value change. Unstandardized estimates before the slash are for the CG and following the slash for the IG. The parameter estimates are corrected for measurement error. Indicator variables are intentionally left out. *p < 0.05, **p < 0.01, ***p < 0.001 (two-tailed *p*-value).

Effects of the intervention were investigated using a multigroup latent change score model (LCSM) that is an extension of the traditional LCSM for between-group comparisons (i.e., non-invariance) of the model parameters (McArdle and Nesselroade, 1994; Kievit et al., 2018). An LCSM is a latent structural equation model for investigating interindividual differences in intraindividual change (Geiser, 2013). These models (see Figure 3) mimic Equation (1),

State 2 = 1 × State 1 + 1 × (State 2 – State 1)(1)

where State 2 (i.e., the post-test) is treated as a function of State 1 (i.e., the pre-test) and the difference between pre-test and post-test. Compared to autoregressive models, where the change is modeled only indirectly via residuals, LCSMs are part of the latent-state-model class and allow obtaining mean and variance estimates for the latent pre-test variable and latent difference variable (State 2 – State 1) directly. Different hypotheses can be tested: (1) the hypothesis of no mean differences over time (μ_Δ_ = 0); (2) the hypothesis of no interindividual differences in the rates of change (σ_Δ_^2^ = 0); (3) the hypothesis of no covariance between the latent baseline variable and latent difference variable (COV[State 1, State 2 – State 1] = 0) (McArdle and Nesselroade, 1994; McArdle, 2009); (4) the explanation of interindividual differences in the initial level (State1) or change scores (State2 – State1) by covariates (Geiser, 2013).

Across-time and between-group assumptions regarding the equality of the factor structure (configural invariance), the same meaning of indicators (metric invariance), and additionally, the intercepts (scalar invariance) need to be ensured for meaningful comparisons in latent mean scores (Steinmetz, 2018). Based on the sample size, we used item parcels as indicator variables for the longitudinally assessed utility-value construct (Little et al., 2002). As shown in Tables B1–B3 (Appendix B), scalar invariance between groups and across time was established.

To identify the mean of the latent pre-test variable and the mean of the latent difference variable, we used the “marker method” (Steinmetz, 2018). We consider Equation (2),

X̅_i_ = τ_i_ + λ_i_ × κ + ε_i_(2)

where τ_i_ defines the intercept, λ_i_ the factor loading, and κ the mean of the latent variable. As E(ε_i_) = 0, ε_i_ can be omitted. By fixing the intercept of the reference indicator (or “marker variable”) to “0,” it follows that the mean of the latent variable is identified by the mean (X̅_i_) of the reference indicator as the factor loading is set to “1” for identification (Geiser, 2013). The mean of the latent difference variable indicates the change from the pre-test to the post-test, and the *value of p* indicates whether the estimate is significantly different from zero. Cohen’s (1988) guidelines for effect sizes of the *d* family were considered, with values of 0.20 indicating small effects, 0.50 indicating moderate effects, and 0.80 indicating large effects. We computed an effects size (*d*_ppc2_), taking pre-test scores into account (Morris, 2008). To test for associations between the latent baseline and difference variable and emotional cost (RQ 2), we computed a conditional multigroup LCSM.

### 2.5. Model estimation and fit evaluation

We evaluated the model fit using the *χ*^2^ statistic, the comparative fit index (CFI), the root mean square error of approximation (RMSEA), and the standardized root mean square residual (SRMR). Hu and Bentler’s (1999) cutoff criteria for model fit indices were applied. Therefore, CFI values ≥0.90 and ≥ 0.95 were considered acceptable or excellent regarding the model fit. An RMSEA of less than 0.06 was considered a good fit, whereas Little (2013), in turn, argues that values of 0.05–0.08 reflect an acceptable fit in longitudinal structural equation modeling. SRMR values of less than 0.08 are considered a good fit, although less is known regarding their performance in longitudinal studies. All models were estimated using M*plus* 8.6 (Muthén and Muthén, 1998–2021) using the maximum likelihood estimator. We used Mardia’s skewness and kurtosis test implemented in M*plus* to investigate whether the maximum likelihood estimator was suitable for estimating the model parameters despite our small sample. We could retain the null hypothesis either for skew (*p* = 0.080) or Kurtosis (*p* = 0.480), indicating that our dependent variables were multivariate normally distributed. Missing data were addressed by full information maximum likelihood (FIML), as appearance was completely random (Little’s MCAR test: χ^2^[10] = 12.63, *p* = 0.245). The FIML approach has been shown to work well even with 50% of missing data and is preferable over listwise deletion with potential higher bias (Enders, 2010).

**Table 1:** Descriptive statistics, sample items, intercorrelations, and composite reliability of study variables.

Variable	Sample item	IG			CG			Correlations		
		*M*	*SD*	ρ	*M*	*SD*	ρ	1.	2.	3.
Utility value	Good knowledge of reflecting on teaching and learning will help me in my future job.									
1. Pre-test		3.13	0.56	0.882	3.33	0.51	0.855	–	0.116	−0.570***
2. Post-test		3.46	0.43	0.834	3.33	0.49	0.827	0.392*	–	0.086
3. Emotional cost (pre-test)	When I deal with reflections, I get annoyed.	1.64	0.61	0.893	1.51	0.45	0.779	−0.550***	−0.393**	–

All variables were measured on a four-point Likert scale. Correlation coefficients listed above the diagonal are for the IG; below the diagonal for the CG. *p ≤ 0.05, **p ≤ 0.01, ***p ≤ 0.001. Reliability estimates are based on previously conducted confirmatory factor analyses.

## 3. Results

### 3.1. *Post-hoc* power analysis

We tested the probability of the implemented hypothesis test to find an effect, given that differences in the population exist, using G*Power 3.1 (Faul et al., 2007). According to Cohen (1992), a power of at least 80% has to be achieved to draw meaningful inferences. We set input parameters for a one-tailed independent samples *t*-test (α = 0.05) and investigated power for medium effect size (Cohen’s *d* = 0.5). The input parameters reflect a notable meta-analysis that found an average effect size of Cohen’s *d* = 0.39 for interventions based on SEVT and Cohen’s *d* = 0.49 for motivational interventions in general (Lazowski and Hulleman, 2016). The *post-hoc* power analysis revealed a power of 80.6%.

### 3.2. Changes in utility values

Descriptive statistics, correlations, and reliabilities are shown in Table 1.^1^ We estimated models where we identified latent mean scores for both groups (marker method). The multigroup LCSM had eight degrees of freedom due to the invariance constraints on factor structure, factor loadings, and item intercepts and showed an excellent fit to the data (*χ*^2^[8] = 7.71, *p* = 0.462, CFI = 1.00, RMSEA = 0.000, 90% CI [0.000, 0.160], SRMR = 0.076). Figure 3 shows the models with coefficients respective for groups. We found significant increases after the writing assignment for the IG (*M* = 0.27, *SE* = 0.13, *p* = 0.030) following a latent baseline score of 2.91 (*SE* = 0.08, *p* < 0.001). In contrast, a pre-test score of 3.05 (*SE* = 0.08, *p* < 0.001) in the CG was followed by a non-significant increase (*M* = 0.05, *SE* = 0.08, *p* = 0.544). The change for the IG was significantly higher than for the CG (*t*[100] = 2.69, *p* = 0.008, Cohen’s *d*_ppc2_ = 0.52).^2^ Differences in pre-test scores turned out to be non-significant (*t*[100] = −1.68, *p* = 0.096). The combination of a positive mean for the latent difference variable and a negative covariance in the IG (covariance = −0.17, *SE* = 0.08, *p* = 0.024) indicates that individuals with lower pre-test utility values showed higher increases after the intervention, whereas participants with higher pre-test scores showed lower increases. In the CG, the covariance of −0.07 turned out to be non-significant (*SE* = 0.05, *p* = 0.136). For both groups, we found significant interindividual differences in the pre-test scores (IG: variance = 0.20, *SE* = 0.08, *p* = 0.009; CG: variance = 0.16, *SE* = 0.07, *p* = 0.022). In contrast, only the variance of the difference variable in the IG (variance = 0.24, *SE* = 0.12, *p* = 0.037) indicated significant interindividual differences in student teachers’ intraindividual change rates, whereas CG participants showed a rather low variability in the rate of change (variance = 0.10, *SE* = 0.06, *p* = 0.104). None of the between-group comparisons in the parameter estimates yielded statistically significant differences. Constraining the latent-variable variances (Δχ^2^[2] = 1.45, *p* = 0.485, ΔCFI = 0.000, ΔRMSEA = 0.000) or covariances (Δχ^2^[3] = 1.87, *p* = 0.601, ΔCFI = 0.000, ΔRMSEA = 0.000), therefore, did not indicate a worse model fit, whereas the SRMR continuously increased (model with equal variances + covariances: 0.164).

### 3.3. Predicting utility-value change

To answer RQ 2, we regressed the latent baseline and latent difference variable on emotional cost at the pre-test. The conditional multigroup LCSM showed an overall acceptable fit to the data [*χ*^2^(46) = 62.10, *p* = 0.057, CFI = 0.936, RMSEA = 0.083, 90% CI (0.000, 0.132), SRMR = 0.122]. As depicted in Figure 3, we found that emotional cost had a significant negative effect on baseline levels (IG: β = −0.43, *SE* = 0.12, *p* < 0.001; CG: β = −0.59, *SE* = 0.18, *p* = 0.001). The effect on the latent difference variable, however, was positive and only significant for the IG (β = 0.40, *SE* = 0.19, *p* = 0.040) but not for the CG (β = 0.08, *SE* = 0.19, *p* = 0.666). The conditional latent change score in the IG remained positive and significant (*M* = 0.21, *SE* = 0.10, *p* = 0.031; variance = 0.15, *SE* = 0.07, *p* = 0.025). Equality constraints on the unstandardized regression parameters did not lead to a substantially worse model fit (Δχ^2^[3] = 4.65, *p* = 0.199, ΔCFI = −0.006, ΔRMSEA = 0.001). However, the SRMR (ΔSRMR = 0.069) provided evidence for misfit. Scripts and outputs for all analyses described in the results section can be obtained from the Open Science Framework.^3^

## 4. Discussion

Student teachers’ utility values have not been the target of systematic interventions to date. However, their implications could influence a wide range of studies. Against the backdrop of the claim that video-based learning “does not automatically activate emotional-motivational processes” (Kleinknecht and Schneider, 2013, p. 21), this study had two aims: First, to investigate utility-value change if student teachers’ interpretations of experiences are systematically harnessed. Second, to consider the extent to which negatively considered task values accompanying video-based tasks, in which own teaching sequences are analyzed, influence motivational developments within the intervention. Crafted on SEVT (Eccles and Wigfield, 2020) and the seldom-regarded component of learners’ interpretations of experiences, we investigated our research questions using a pre-test-post-test control group design after the end of a teaching practicum, in which student teachers’ lessons were either videotaped (IG) or protocoled (CG).

### 4.1. Utility-value change

Regarding the first aim of our study, the results were consistent with our hypotheses. Utility values were promoted with a medium effect size compared to CG (confirming *Hypothesis 1.1*) while mirroring previous interventions and their effect sizes with a similar magnitude (see Lazowski and Hulleman, 2016). Reasons for learning might not be obvious (Wentzel and Brophy, 2014), even though videos of one’s teaching might enhance student teachers’ learning during a teaching practicum (Charalambous et al., 2022). Our study helped student teachers identify the utility of reflections on teaching and learning better than a protocol-based reflection with an unstructured writing assignment. Again, it must be referred to the promises and pitfalls of video application in teacher education. A vital review has pointed out that video-based reflections necessitate scaffolding to make them an effective learning experience for student teachers (Gaudin and Chaliès, 2015). Therefore, instructional guidance must appear highly structured to make video truly an opportunity to overcome the theory-practice gap (Korthagen, 2010). Plenty of studies (e.g., Brouwer et al., 2017) delivered structured viewing guides to their participants and ultimately led to enhanced targeted teaching behaviors such as dimensions of teaching quality (e.g., cognitive activation) for either student or in-service teachers. Research has agreed that facilitators (e.g., cooperating teachers, teacher educators, etc.) play a crucial role in guiding student teachers’ focus on the events in the classroom and topics that matter (Seidel and Stürmer, 2014; Goldberg et al., 2021). If this aspect is not ensured, student teachers will feel overwhelmed by the many aspects to simultaneously reflect on and perceive less utility (Tripp and Rich, 2012). Even though this regards video-based tools, it should also enrich our understanding of learners’ characteristics in a wide range of interventions. To this end and next to implications arising from motivational literature, the writing assignment in our study can be considered as working on student teachers’ theory-practice relations in a way that a motivationally relevant experience resulted (*cf.* Hulleman and Harackiewicz, 2021). Courses in teacher education that make use of video provide *representations of practice* (Grossman et al., 2009). The degree of authenticity or extent to which an authentic representation is given is not merely the video. A video of one’s teaching is more or less proximal to the teaching practices, although it attempts to offer a more authentic learning opportunity for reflections on teaching and learning (McDonald et al., 2013). One way of moving forward to more intense and authentic experiences is to encourage student teachers to take a critical stance toward their achievement-related experiences with videos, to discover how they want to improve their teaching based on their experiences, and to let them seek relationships with the professional practice that teacher educators struggle to implement by task design (Kleinknecht and Schneider, 2013; Brouwer et al., 2017; Kang and van Es, 2019). The findings of this intervention study support the attempt that student teachers may be in the position to learn from video-based reflection of their own teaching.

Another aspect is related to the substantial variability in student teachers’ latent difference scores. Lower scores before the intervention covaried with higher change scores or vice versa (confirming *Hypothesis 1.2*). In the context of utility-value-like interventions, it is less surprising that especially those with a history of poor achievement, higher perceptions of cost, and related lower utility values benefit. Therefore, the study by Canning et al. (2018) is noteworthy as they critically investigated the role of timing. Lower-performing students benefitted more regarding course grades and showed a tendency concerning enrollment in the second course when the writing assignment was implemented in the first unit compared to the last. The opposite was true for higher-performing students. In our intervention, the writing assignments on student teachers’ achievement-related experiences were implemented at the end of the semester. Therefore, they did not mirror this aspect of Canning et al.’s findings. Altogether, these findings beg implications for video-based interventions. Thus, identifying predictors for changes in utility values, as a study by Canning et al. (2019) did, will help to verify the findings and aid subsequent attempts in considering student teachers’ psychological characteristics.

### 4.2. The role of emotional cost in utility-value change

Regarding the second aim of our study, the findings were again consistent with the hypothesis. Implementing a writing assignment covering student teachers’ perceptions of their achievement-related experiences influenced structural relations in the way that emotional cost predicted student teachers’ utility-value change positively after the writing assignment (confirming *Hypothesis 2*). Apart from past explanations—studies comparing (student) teachers watching videos of their own vs. others’ teaching (e.g., Kleinknecht and Schneider, 2013; Chan et al., 2018)—a more processual view could be held. Outcome-focused explanations highlighted discrepancies between expectations and the videotaped result as a possible trigger for negative emotions (Chan et al., 2018). However, it could be assumed that own teaching sequences will also activate mental representations of teacher-student relationships. According to a well-known relational model (Spilt et al., 2011), the association between classroom events (e.g., student misbehavior) and teacher well-being is mediated by mental representations of the teacher-student relationship (i.e., the degree of closeness and conflict). Video as an immersive experience shows merits in enabling student teachers to reflect retrospectively without situational pressure to act at home or in a video club with peers (van Es, 2012; Brouwer et al., 2017; Charalambous et al., 2018, 2022). However, there are also pitfalls. Repeatedly viewed adverse classroom events could trigger negative emotions, at least partially mediated by teacher-student relationships. This perspective could guide future research.

After adjusting for baseline emotional cost, the latent change score in the IG remained significant and positive. In sum, the findings of the conditional LCSM underpin the efficacy of the writing assignment for the often-mentioned difficulties in the implementation of video-based tools (Chan et al., 2018). This study helped incapacitate troublesome characteristics of video-based tasks and reflected parts of Rosenzweig et al.’s (2020) intervention materials. It should be considered that much research has pointed to the negative influence of cost on the learning outcome as well as other subjective task values and expectancies (Eccles, 2005; Pekrun, 2006; Flake et al., 2015). For the latent pre-test utility values, this was the case as expected. For the latent difference scores, the results point out how the intervention helped student teachers with higher perceptions of cost after the teaching practicum, in turn stressing the need for researchers and teacher educators to reflect carefully on costly characteristics in video-based learning.

Our intervention provided a wise approach to harnessing and fostering emotional-motivational processes in a video-based task. Thus, video-based interventions can be supportive of situating student teachers’ reflections; however, doing such tasks may also be a troublesome experience with increasing negative emotions (Kleinknecht and Schneider, 2013). Therefore, interventions during a teaching practicum need to be carefully designed to be a successful and high-leverage learning opportunity.

### 4.3. Limitations

Although the present study overcame several issues related to important previous exploratory research (e.g., Chan et al., 2018), some limitations should be taken into account. First, the study made it possible to compare the effects of video and a motivational intervention with a larger sample size than previous studies were able to accomplish. However, the study is—as many video-based studies and interventions in teaching practicums—largely depended on the context (e.g., countries and characteristics such as the duration). So-called “Many Labs”-projects would improve the confidence concerning the existence of effects from video-based studies and thus aid in overcoming their assumed situated character and dependence on aspects of the data collection (see Klein et al., 2014). A second restriction is that we considered item parcels as indicator variables due to the limited sample size (Little et al., 2002). As this is an established method to make structural equation models less complex and has been applied in similar research in teacher education (e.g., Gold et al., 2017), we achieved satisfactory model fit indices. However, risks such as estimation bias and model misspecification must be considered carefully (Matsunaga, 2008). Therefore, confirmatory factor analyses were conducted by Dehne et al. (2018) in a previous study, ensuring the assumed dimensionality with the original number of items. Furthermore, none of the between-group comparisons for the structural parameters (i.e., variances, covariances, and regression parameters) yielded significantly higher/lower overall estimates for the IG. As a result, comparisons between both groups have to be taken as tendencies. However, it has to be remarked that a couple of non-significant estimates (variance, covariance, and regression of the latent difference variable on emotional cost) contrasted statistically significant estimates in the IG. Finally, one limitation of the study is that student teachers’ essays were not examined using qualitative analyses. In most utility-value interventions, the written essays were not systematically investigated. However, exploratory research by Canning et al. (2019) has pointed to the importance of considering the quality of students’ essay-based writing assignments. As this was beyond the scope of the present research, this perspective could guide future avenues of research.

### 4.4. Implications and future directions for research

This study has practical implications for the use of video in teacher education. Student teachers appreciate the systematic and contextualized use of video, and it helps them to identify the utility of reflecting on teaching and learning for their future careers. However, the use of video also has emotional costs, and not all student teachers benefit from it. That means teacher educators should carefully select video-based tasks or other digital tools and be aware of their consequences for student teachers’ learning (*cf.* Kang and van Es, 2019). More immersive tools, such as virtual reality (VR), have become increasingly available and could enable a better opportunity regarding emotional-motivational processes. Two studies (Huang et al., 2022; Richter et al., 2022) compared VR with video in teacher education. Richter et al. (2022) concluded from their quasi-experimental study that reflective learning processes were equally triggered in both conditions. In contrast, reflection-related self-efficacy was only fostered in the VR group. Huang et al. (2022) conducted a pre-registered experiment and found favorable results for the VR condition in terms of interest and self-efficacy in classroom management. However, the extraneous cognitive load was also higher for the VR task, which would again require approaching negative task-related aspects. As a consequence, future research should take utility values and the role of emotional cost into consideration. These important predictors of student teachers’ learning have yet to be investigated in the context of VR or other immersive technologies.

## 5. Conclusion

Video-based tools in teacher education provide a complex learning opportunity, and student teachers may need additional support to achieve the learning objectives. This support includes material artifacts as instructional tools to augment or prearrange videos for student teachers’ learning. The study describes the successful implementation of a learning opportunity that orchestrated student teachers’ interpretations of experiences in order to foster emotional-motivational beliefs. By critically investigating the role of psychological characteristics in a video-based intervention, this study adds value to the fields of educational psychology and teacher education as it addresses the less comprehensively studied parts of the SEVT model and the cost component. In future research, both utility values and emotional cost need consideration because they are critical psychological characteristics of participants accomplishing a video-based task. Promoting student teachers’ motivation to reflect routinely is a professional task for teacher education (Kunter, 2013). Continuous use of learned content is at the heart of the teaching profession and is relevant in any educational setting. For this purpose, student teachers need to identify its utility value.

## Data availability statement

The raw data supporting the conclusions of this article will be made available by the authors, without undue reservation and based on German data protection law.

## Ethics statement

The study was reviewed and approved by the Thuringian Ministry of Education, Youth and Sport, Erfurt, Germany. Written informed consent to participate in this study was provided by the participants.

## Author contributions

MD and AG conceptualized the idea, contributed to the study design, and collected data. MD was responsible for data curation, executing the formal analysis, writing, revising, and editing the original draft, and visualizing research findings. AG administered the project, acquired funding, and reviewed and approved the submitted manuscript. All authors contributed to the article and approved the submitted version.

## Funding

The study was funded by the Federal Ministry of Education and Research (project number 01JA1808). Furthermore, we acknowledge support by the German Research Foundation (project number 512648189) and the Open Access Publication Fund of the Thueringer Universitaets - und Landesbibliothek Jena.

## Conflict of interest

The authors declare that the research was conducted in the absence of any commercial or financial relationships that could be construed as a potential conflict of interest.

## Publisher’s note

All claims expressed in this article are solely those of the authors and do not necessarily represent those of their affiliated organizations, or those of the publisher, the editors and the reviewers. Any product that may be evaluated in this article, or claim that may be made by its manufacturer, is not guaranteed or endorsed by the publisher.

## Footnotes

^1^In pre-analysis, we tested for changes from the beginning (background survey) to the end of the teaching practicum (pre-test). The multigroup LCSM fitted the data well (χ^2^[8] = 10.51, *p* = 231, CFI = 0.977, RMSEA = 0.071, 90% CI (0.000, 0.173), SRMR = 0.057]. Neither in the IG (*M* = 2.96, variance = 0.19, *M* = −0.06, variance = 0.28) nor in the CG (*M* = 3.03, variance = 0.20, *M* = 0.06, variance = 0.18), significant changes in utility values occurred across the teaching practicum.

^2^We refer detailed to problems associated with the reference-group method for the identification of mean structures in multigroup LCSM under the following link on the Open Science Framework: https://osf.io/8fm53/?view_only=48370a3ef7624402a1c38b1564f36402.

^3^https://osf.io/8fm53/?view_only=48370a3ef7624402a1c38b1564f36402

## References

[ref1] Backfisch I. Lachner A. Stürmer K. Scheiter K. (2021). Variability of teachers’ technology integration in the classroom: a matter of utility! Comp. Educ. 166:104159. doi: 10.1016/j.compedu.2021.104159

[ref2] Bardach L. Klassen R. M. Perry N. E. (2022). Teachers’ psychological characteristics: do they matter for teacher effectiveness, teachers’ well-being, retention, and interpersonal relations? An integrative review. Educ. Psychol. Rev. 34, 259–300. doi: 10.1007/s10648-021-09614-9

[ref3] Beck C. Kosnik C. (2002). Components of a good practicum placement: pre-service teacher perceptions. Teach. Educ. Quart. 29, 81–98.

[ref4] Brouwer N. Besselink E. Oosterheert I. (2017). The power of video feedback with structured viewing guides. Teach. Teach. 66, 60–73. doi: 10.1016/j.tate.2017.03.013

[ref5] Canning E. A. Harackiewicz J. M. Priniski S. J. Hecht C. A. Tibbetts Y. Hyde J. S. (2018). Improving performance and retention in introductory biology with a utility-value intervention. J. Educ. Psychol. 110, 834–849. doi: 10.1037/edu0000244, PMID: 30294006 PMC6168083

[ref6] Canning E. A. Priniski S. J. Harackiewicz J. M. (2019). Unintended consequences of framing a utility-value intervention in two-year colleges. Learn. Instr. 62, 37–48. doi: 10.1016/j.learninstruc.2019.05.001

[ref7] Chan K. K. H. He C. Ng R. C. K. Leung J. S. C. (2018). Student teachers’ emotions when watching their own videos and those of their peers. J. Prof. Capit. Community 3, 192–211. doi: 10.1108/JPCC-12-2017-0031

[ref8] Charalambous C. Y. Philippou S. Olympiou G. (2018). Reconsidering the use of video clubs for student-teachers learning during field placement: lessons drawn from a longitudinal multiple case study. Teach. Teach. 74, 49–61. doi: 10.1016/j.tate.2018.04.002

[ref9] Charalambous C. Y. Philippou S. Olympiou G. Georgiou K. (2022). Experimenting with enablers and extenders to support ambitious teaching in mathematics: a video-club case study of student teachers during their field placement. Teach. Teach. 119:103874. doi: 10.1016/j.tate.2022.103874

[ref10] Cohen J. (1988). Statistical power analysis for the behavioral sciences. Mahwah, NJ: Lawrence Erlbaum Associates.

[ref11] Cohen J. (1992). A power primer. Psychol. Bull. 112, 155–159. doi: 10.1037/0033-2909.112.1.155, PMID: 19565683

[ref12] Dehne M. Klaß S. Gröschner A. (2018). Veränderung motivationaler Orientierungen im Praxissemester: Eine videobasierte Studie auf basis der Erwartungs-wert-Theorie [changes in motivational orientations during pre-service teacher practicum: a video-based study based on expectancy-value theory]. Lehrerbildung auf dem Prüfstand 11, 109–130.

[ref13] Eccles J. S. (2005). “Subjective task value and the Eccles et al. model of achievement-related choices” in Handbook of competence and motivation. eds. Elliot A. J. Dweck C. S. (New York: Guilford Press), 105–121.

[ref14] Eccles J. S. Wigfield A. (2002). Motivational beliefs, values, and goals. Ann. Rev. Psych. 53, 109–132. doi: 10.1146/annurev.psych.53.100901.135153 11752481

[ref15] Eccles J. S. Wigfield A. (2020). From expectancy-value theory to situated expectancy-value theory: a developmental, social cognitive, and sociocultural perspective on motivation. Contemp. Educ. Psychol. 61:101859. doi: 10.1016/j.cedpsych.2020.101859

[ref16] Enders C. K. (2010). Applied missing data analysis. New York: Guilford Press.

[ref17] Faul F. Erdfelder E. Lang A.-G. Buchner A. (2007). G*power 3: a flexible statistical power analysis program for the social, behavioral, and biomedical sciences. Behav. Res. Meth. 39, 175–191. doi: 10.3758/BF03193146, PMID: 17695343

[ref18] Flake J. K. Barron K. E. Hulleman C. McCoach B. D. Welsh M. E. (2015). Measuring cost: the forgotten component of expectancy-value theory. Contemp. Educ. Psychol. 41, 232–244. doi: 10.1016/j.cedpsych.2015.03.002

[ref19] Gaspard H. Dicke A.-L. Flunger B. Brisson B. M. Häfner I. Nagengast B.. (2015a). Fostering adolescents’ value beliefs for mathematics with a relevance intervention in the classroom. Dev. Psychol. 51, 1226–1240. doi: 10.1037/dev0000028, PMID: 26192044

[ref20] Gaspard H. Dicke A.-L. Flunger B. Schreier B. Häfner I. Trautwein U.. (2015b). More value through greater differentiation: gender differences in value beliefs about math. J. Educ. Psychol. 107, 663–677. doi: 10.1037/edu0000003

[ref21] Gaspard H. Parrisius C. Piesch H. Kleinhansl M. Wille E. Nagengast B.. (2021). The potential of relevance interventions for scaling up: a cluster-randomized trial testing the effectiveness of a relevance intervention in math classrooms. J. Educ. Psychol. 113, 1507–1528. doi: 10.1037/edu0000663

[ref22] Gaudin C. Chaliès S. (2015). Video viewing in teacher education and professional development: a literature review. Educ. Res. Rev. 16, 41–67. doi: 10.1016/j.edurev.2015.06.001

[ref23] Geiser C. (2013). Data analysis with Mplus. New York: Guilford Press.

[ref24] Gold B. Hellermann C. Holodynski M. (2017). Effekte videobasierter Trainings zur Förderung der Selbstwirksamkeitsüberzeugungen über Klassenführung im Grundschulunterricht [Effects of video-based trainings for promoting self-efficacy in elementary classroom management]. Z. Erzieh. 20, 115–136. doi: 10.1007/s11618-017-0727-5

[ref25] Goldberg P. Schwerter J. Seidel T. Müller K. Stürmer K. (2021). How does learners’ behavior attract preservice teachers’ attention during teaching? Teach. Teach. 97:103213. doi: 10.1016/j.tate.2020.103213

[ref26] Goldman R. (2007). “Video representations and the perspectivity framework: epistemology, ethnography, evaluation, and ethics” in Video research in the learning sciences. eds. Goldman R. Pea R. Barron B. Derry S. J. (Mahwah, NJ: Lawrence Erlbaum), 383–395.

[ref27] Grossman P. Compton C. Igra D. Ronfeldt M. Shahan E. Williamson P. W. (2009). Teaching practice: a cross-professional perspective. Teach. Coll. Rec. 111, 2055–2100. doi: 10.1177/016146810911100905

[ref28] Harackiewicz J. M. Priniski S. J. (2018). Improving student outcomes in higher education: the science of targeted intervention. Ann. Rev. Psychol. 69, 409–435. doi: 10.1146/annurev-psych-122216-011725, PMID: 28934586 PMC6211287

[ref29] Harackiewicz J. M. Rozek C. S. Hulleman C. S. Hyde J. M. (2012). Helping parents to motivate adolescents in mathematics and science: an experimental test of a utility value intervention. Psychol. Sci. 23, 899–906. doi: 10.1177/0956797611435530, PMID: 22760887

[ref31] Huang Y. Richter E. Kleickmann T. Richter D. (2022). Comparing video and virtual reality as tools for fostering interest and self-efficacy in classroom management: results of a pre-registered experiment. Brit. J. Educ. Tech. 54, 467–488. doi: 10.1111/bjet.13254

[ref30] Hu L. Bentler P. M. (1999). Cutoff criteria for fit indexes in covariance structure analysis: conventional criteria versus new alternatives. Struct. Equ. Model. 6, 1–55. doi: 10.1080/10705519909540118

[ref001] Hulleman C. S. Harackiewicz J. M. (2009). Promoting interest and performance in high school science classes. Science 326, 1410–1412. doi: 10.1126/science.1177067 19965759

[ref32] Hulleman C. S. Harackiewicz J. M. (2021). “The utility-value intervention” in Handbook of wise interventions: How social psychology can help people change. eds. Walton G. M. Crum A. J. (New York: Guilford Press), 100–125.

[ref33] Kang H. van Es E. A. (2019). Articulating design principles for productive use of video in preservice education. J. Teach. Educ. 70, 237–250. doi: 10.1177/0022487118778549

[ref34] Kievit R. A. Brandmaier A. M. Ziegler G. van Harmelen A.-L. de Mooij S. M. M. Moutoussis M.. (2018). Developmental cognitive neuroscience using latent change score models: a tutorial and applications. Dev. Cogn. Neurosci. 33, 99–117. doi: 10.1016/j.dcn.2017.11.007, PMID: 29325701 PMC6614039

[ref35] Klassen R. M. Kim L. E. Rushby J. V. Bardach L. (2020). Can we improve how we screen applicants for initial teacher education? Teach. Teach. 87:102949. doi: 10.1016/j.tate.2019.102949

[ref36] Klein R. A. Ratliff K. A. Vianello M. Adams R. B. Jr. Bahník Š. Bernstein M. J.. (2014). Investigating variation in replicability: a “many labs” replication project. Soc. Psychol. 45, 142–152. doi: 10.1027/1864-9335/a000178

[ref37] Kleinknecht M. Gröschner A. (2016). Fostering preservice teachers’ noticing with structured video feedback: results of an online– and video-based intervention study. Teach. Teach. 59, 45–56. doi: 10.1016/j.tate.2016.05.020

[ref38] Kleinknecht M. Schneider J. (2013). What do teachers think and how do they feel when they analyze videos of themselves teaching and of other teachers teaching? Teach. Teach. 33, 13–23. doi: 10.1016/j.tate.2013.02.002

[ref39] Koehler M. J. Yadav A. Phillips M. M. Cavazos-Kottke S. C. (2005). What is video good for? Examining how media and story genre interact. J. Educ. Multim. Hyperm. 14, 249–272.

[ref40] König J. Santagata R. Scheiner T. Adleff A. Yang X. Kaiser G. (2022). Teacher noticing: a systematic literature review of conceptualizations, research designs, and findings on learning to notice. Educ. Res. Rev. 36:100453. doi: 10.1016/j.edurev.2022.100453

[ref41] Korthagen F. A. J. (2010). Situated learning theory and the pedagogy of teacher education: towards an integrative view of teacher behavior and teacher learning. Teach. Teach. 26, 98–106. doi: 10.1016/j.tate.2009.05.001

[ref42] Kunter M. (2013). “Motivation as an aspect of professional competence: research findings on teacher enthusiasm” in Cognitive activation in the mathematics classroom and professional competence of teachers. eds. Kunter M. Baumert J. Blum W. Klusmann U. Krauss S. Neubrand M. (New York: Springer), 273–289. doi: 10.1007/978-1-4614-5149-5_13

[ref43] Lazowski R. A. Hulleman C. S. (2016). Motivation interventions in education: a Meta-analytic review. Rev. Educ. Res. 86, 602–640. doi: 10.3102/0034654315617832

[ref44] Little T. D. (2013). Longitudinal structural equation modeling. New York: Guilford.

[ref45] Little T. D. Cunningham W. A. Shahar G. Widaman K. F. (2002). To parcel or not to parcel: exploring the question, weighing the merits. Struct. Equ. Model. 9, 151–173. doi: 10.1207/S15328007SEM0902_1

[ref46] Matsunaga M. (2008). Item parceling in structural equation modeling: a primer. Communic. Meth. Meas. 2, 260–293. doi: 10.1080/19312450802458935

[ref47] McArdle J. J. (2009). Latent variable modeling of differences and changes with longitudinal data. Ann. Rev. Psychol. 60, 577–605. doi: 10.1146/annurev.psych.60.110707.163612 18817479

[ref48] McArdle J. J. Nesselroade J. R. (1994). “Using multivariate data to structure developmental change” in Life-span developmental psychology: Methodological contributions. eds. Cohen S. H. Reese H. W. (Mahwah, NJ: Lawrence Erlbaum Associates, Inc), 223–267.

[ref49] McDonald M. Kazemi E. Kavanagh S. S. (2013). Core practices and pedagogies of teacher education: a call for a common language and collective activity. J. Teach. Educ. 64, 378–386. doi: 10.1177/0022487113493807

[ref50] Moreno R. Valdez A. (2007). Immediate and delayed effects of using a classroom case exemplar in teacher education: the role of presentation format. J. Educ. Psychol. 99, 194–206. doi: 10.1037/0022-0663.99.1.194

[ref51] Morris S. B. (2008). Estimating effect sizes from pre-test-post-test-control group designs. Org. Res. Meth. 11, 364–386. doi: 10.1177/1094428106291059

[ref52] Muthén L.K. Muthén B.O. (1998–2021). Mplus user’s guide (8th ed.). Los Angeles, CA: Muthén and Muthén.

[ref53] Nickl M. Sommerhoff D. Böheim R. Ufer S. Seidel T. (2023). Fostering pre-service teachers’ assessment skills in a video simulation. Zeitschrift für Pädagogische Psychologie. doi: 10.1024/1010-0652/a000362

[ref54] O’Connor C. Michaels S. Chapin S. Harbaugh A. G. (2017). The silent and the vocal: participation and learning in whole-class discussion. Learn. Instr. 48, 5–13. doi: 10.1016/j.learninstruc.2016.11.003

[ref55] Part R. Perera H. N. Marchand G. C. Bernacki M. L. (2020). Revisiting the dimensionality of subjective task value: towards clarification of competing perspectives. Contemp. Educ. Psychol. 62:101875. doi: 10.1016/j.cedpsych.2020.101875

[ref56] Pekrun R. (2006). The control-value theory of achievement emotions: assumptions, corollaries, and implications for educational research and practice. Educ. Psychol. Rev. 18, 315–341. doi: 10.1007/s10648-006-9029-9

[ref57] Perez T. Cromley J. G. Kaplan A. (2014). The role of identity development, values, and costs in college STEM retention. J. Educ. Psychol. 106, 315–329. doi: 10.1037/a0034027

[ref58] Piesch H. Häfner I. Gaspard H. Flunger B. Nagengast B. Harackiewicz J. M. (2019). Helping parents support adolescents’ career orientation: effects of a parent-based utility-value intervention. Unterrichtswissenschaft 47, 271–293. doi: 10.1007/s42010-018-0024-x

[ref59] Prilop C. N. Weber K. E. Kleinknecht M. (2019). How digital reflection and feedback environments contribute to pre-service teachers’ beliefs during a teaching practicum. Stud. Educ. Eval. 62, 158–170. doi: 10.1016/j.stueduc.2019.06.005

[ref60] Richards J. Altshuler M. Sherin B. L. Sherin M. G. Leatherwood C. J. (2021). Complexities and opportunities in teachers’ generation of videos from their own classrooms. Learn. Cult. Soc. Inter. 28:100490. doi: 10.1016/j.lcsi.2021.100490

[ref61] Richter E. Hußner I. Huang Y. Richter D. Lazarides R. (2022). Video-based reflection in teacher education: comparing virtual reality and real classroom videos. Comp. Educ. 190:104601. doi: 10.1016/j.compedu.2022.104601

[ref62] Rosenzweig E. Q. Wigfield A. Hulleman C. S. (2020). More useful or not so bad? Examining the effects of utility value and cost reduction interventions in college physics. J. Educ. Psychol. 112, 166–182. doi: 10.1037/edu0000370

[ref63] Santagata R. König J. Scheiner T. Nguyen H. Adleff A.-K. Yang X.. (2021). Mathematics teacher learning to notice: a systematic review of studies of video-based programs. ZDM Math. Educ. 53, 119–134. doi: 10.1007/s11858-020-01216-z

[ref64] Seidel T. Stürmer K. Blomberg G. Kobarg M. Schwindt K. (2011). Teacher learning from analysis of videotaped classroom situations: does it make a difference whether teachers observe their own teaching or that of others? Teach. Teach. 27, 259–267. doi: 10.1016/j.tate.2010.08.009

[ref65] Seidel T. Stürmer K. (2014). Modeling and measuring the structure of professional vision in preservice teachers. Am. Educ. Res. J. 51, 739–771. doi: 10.3102/0002831214531321

[ref66] Song Y. Rosenzweig E. Q. Barger M. M. (in press). Disentangling emotional cost, psychological cost, and anxiety in motivation research. Mot. Emo.

[ref67] Spilt J. L. Koomen H. M. Y. Thijs J. T. (2011). Teacher wellbeing: the importance of teacher–student relationships. Educ. Psychol. Rev. 23, 457–477. doi: 10.1007/s10648-011-9170-y

[ref68] Steinmetz H. (2018). “Estimation and comparison of latent means across cultures” in Cross-cultural analysis: Methods and applications. eds. Davidov E. Schmidt P. Billiet J. Meuleman B. (New York: Routledge), 95–126.

[ref69] Teo T. (2009). Modelling technology acceptance in education: a study of pre-service teachers. Comp. Educ. 52, 302–312. doi: 10.1016/j.compedu.2008.08.006

[ref70] Tripp R. T. Rich J. R. (2012). The influence of video analysis on the process of teacher change. Teach. Teach. 28, 728–739. doi: 10.1016/J.TATE.2012.01.011

[ref71] Tschannen-Moran M. Hoy A. W. Hoy W. K. (1998). Teacher efficacy: its meaning and measure. Rev. Educ. Res. 68, 202–248. doi: 10.3102/00346543068002202

[ref72] Vandewaetere M. Desmet P. Clarebout G. (2011). The contribution of learner characteristics in the development of computer-based adaptive learning environments. Comp. Hum. Behav. 27, 118–130. doi: 10.1016/j.chb.2010.07.038

[ref73] van Es E. A. (2012). Examining the development of a teacher learning community: the case of a video club. Teach. Teach. 28, 182–192. doi: 10.1016/j.tate.2011.09.005

[ref74] Vermunt J. D. Endedijk M. D. (2011). Patterns in teacher learning in different phases of the professional career. Learn. Individ. Differ. 21, 294–302. doi: 10.1016/j.lindif.2010.11.019

[ref75] Walton G. M. (2014). The new science of wise psychological interventions. Curr. Direct. Psychol. Sci. 23, 73–82. doi: 10.1177/0963721413512856

[ref76] Weber K. E. Prilop C. N. Kleinknecht M. (2023). Effects of different video– or text-based reflection stimuli on pre-service teachers’ emotions, immersion, cognitive load and knowledge-based reasoning. Stud. Educ. Eval. 77:101256. doi: 10.1016/j.stueduc.2023.101256

[ref77] Wentzel K. R. Brophy J. E. (2014). Motivating students to learn. New York: Routledge

[ref78] Zeichner K. (2010). Rethinking the connections between campus courses and field experiences in college– and university–based teacher education. J. Teach. Educ. 61, 89–99. doi: 10.1177/0022487109347671

