# Supplementary material for: Utility-value change and the role of emotional cost in video-based learning: a matter of student teachers’ interpretation of experience
Source: Front Psychol. 2023 Jul 26;14:1166921. doi: 10.3389/fpsyg.2023.1166921 (PMC10411348; doi:10.3389/fpsyg.2023.1166921)
Supplement: Supplementary file 1 [file Data_Sheet_1.pdf]

Supplementary Material

Utility-value change and the role of emotional cost in video-based learning: A matter of student teachers' interpretation of experience

Appendix A

Table A1

Individual Items of the Utility-Value and Emotional-Cost Scale

Scale	Items
Utility value	Good results in reflecting on teaching and learning can be of great value to me later on.
	Learning to reflect is worthwhile because it improves my job and career chances.
	Good knowledge of reflecting on teaching and learning will help me in my future job.
	Reflections on teaching and learning will be helpful to me in my future practice.
	Being able to reflect well will pay off for my future career.
Emotional cost	I'd rather not reflect because it only worries me.
	When I deal with reflections, I get annoyed.
	Reflecting on teaching and learning is a real burden to me.
	Reflecting on teaching and learning makes me really nervous.

Appendix B

Table B1

Multigroup Invariance Testing Between Groups

Model	Fit-indices of the latent single state models					Difference tests				
	χ^2	*df*	*p*	CFI	RMSEA	$\Delta\chi^2$	Δdf	*p*	ΔCFI	ΔRMSEA
Utility value (pretest)	0.00	0	.000	1.00	.000					
	0.39	1	.534	1.00	.000	0.39	1	.534	.000	.000
Utility value (posttest)	0.00	0	.000	1.00	.000					
	0.16	1	.686	1.00	.000	0.16	1	.686	.000	.000

Note. Fixing both factor loadings at "1" was necessary to identify one-dimensional multigroup models with two indicator variables. Therefore, configural and metric invariant models are statistically equivalent, and the fit indices are reported once. The fit indices in the first position are for the configural/metric invariant model. The fit indices in the second position are for the scalar invariant model.

Table B2

Longitudinal Invariance Testing

Model	Fit-indices of the latent state models					Difference tests				
	χ^2	*df*	*p*	CFI	RMSEA	$\Delta\chi^2$	Δdf	*p*	ΔCFI	ΔRMSEA
Utility value	0.72	1	.397	1.00	.000					
	0.76	2	.683	1.00	.000	0.05	1	.830	.000	.000
	0.79	3	.851	1.00	.000	0.03	1	.860	.000	.000

Note. The fit indices in the first position are for the configural invariant model. The fit indices in the second position are for the metric invariant model. The fit indices listed in the third position are for the scalar invariant model.

Table B3

Multigroup Longitudinal Invariance Testing

Model	Fit-indices of the latent state models					Difference tests				
	χ^2	*df*	*p*	CFI	RMSEA	$\Delta\chi^2$	Δdf	*p*	ΔCFI	ΔRMSEA
Utility value	7.51	6	.277	.975	.070					
	7.71	7	.359	.988	.045	0.21	1	.650	.013	–.025
	7.71	8	.462	1.00	.000	0.00	1	.975	.011	–.045

Note. The fit indices in the first position are for the configural invariant model. The fit indices in the second position are for the metric invariant model. The fit indices listed in the third position are for the scalar invariant model.
